# A case of galactosemia misdiagnosed as cow’s milk intolerance

**DOI:** 10.1186/1824-7288-38-47

**Published:** 2012-09-19

**Authors:** Roberto Della Casa, Carla Ungaro, Emma Acampora, Claudio Pignata, Pietro Vajro, Mariacarolina Salerno, Francesca Santamaria, Giancarlo Parenti

**Affiliations:** 1 Department of Pediatrics, Federico II University, Via S. Pansini 5, 80131 Naples, Italy; 2 Department of Pediatrics, University of Salerno, Salerno, Italy

**Keywords:** Galactosemia, Cow’s milk intolerance, Metabolic disease.

## Abstract

We report on a female patient affected by galactosemia in whom the diagnosis was obscured by the concomitant presence of manifestations suggesting a cow’s milk intolerance. This case exemplifies the problems in reaching a correct diagnosis in patients with metabolic diseases.

## Introduction

The metabolic diseases are a clinically heterogeneous group of genetic disorders characterized by variable involvement of multiple organs and systems. As the clinical presentations of these disorders are often non-specific and may mimic other diseases, it is not uncommon that metabolic patients are not correctly recognized and that there is a substantial delay between onset of clinical manifestations and diagnosis. This is also due to the fact that metabolic diseases are rare disorders, in some cases with an extremely low prevalence, and are poorly known to many physicians.

Here we present a case of galactosemia that exemplifies the difficulties in achieving a correct diagnosis when clinical manifestations are overshadowed by concomitant disorders.

### Case report

FB, a female, was born to non-consanguineous parents. Her mother was affected by epilepsy and assumed anticonvulsants (carbamazepine) throughout pregnancy. She was born at 37 weeks of gestation. Delivery was uneventful.

After birth she was fed with a standard formula. Jaundice became evident in the first few days, with a total bilirubin of 15.5 mg/dl at 4 days, and was treated with phototherapy. At 7 days she was referred to a neonatal intensive care unit of a Hospital in Naples because of poor feeding, recurrent vomiting, and abdominal distention. Routine biochemistry showed the presence of defective coagulation (PT 27%), modest hypertransaminasemia (AST 156 U/L; ALT 188 U/L), and a transient hyperammonemia (202 mcrg/dl) that was not detected in subsequent determinations. Biochemical analyses to detect causes of infectious diseases were negative. Screenings for metabolic disorders (blood gas analysis, plasma acylcarnitine profile by tandem mass spectrometry, plasma amino acids, urinary orotic acid and organic acids, and a screening for congenital defects of glycosylation) were all normal or non-informative. Brain, heart and abdominal ultra-sound scans were normal. An ophthalmoscopy was also normal.

During the period in the intensive care unit she was fed with a low-protein, lactose-free, hydrolysate formula for 9 days, until the age of 16 days, with progressive clinical improvement and complete recovery from the biochemical abnormalities. This formula was chosen in the hypothesis of a cow’s milk intolerance because of the presence of vomiting. Considering the occasional detection of hyperammonemia, the daily protein intake was restricted.

At the age of 16 days the hydrolysate formula was interrupted and a standard formula feeding was re-introduced, still with a reduced intake of proteins. Again, vomiting and anorexia occurred, further supporting the hypopthesis of cow’s milk intolerance. Thus, the same protein hydrolysate formula used earlier was resumed and continued in the following months.

She was discharged from the Hospital at the age of 40 days, with a low-protein hydrolysate formula. At that time routine biochemical analyses, including blood ammonia and coagulation were normal.

She was referred to our Department at the age of 3 months and 20 days for further diagnostic work-up, specifically focused on possible causes of hyperammonemia. During her stay at our Department all biochemical analyses and screenings for metabolic disorders, including urea cycle defects and other possible causes of hyperammonemia, were normal or negative.

She was therefore discharged from our Department with a protein hydrolysate formula, this time with a normal intake of proteins and calories, and followed with periodic admissions to our Day-Hospital.

At the age of 8 months a formula containing cow’s milk proteins was reintroduced. Once again, after 2 weeks the patient presented with anorexia and vomiting and on a follow-up biochemical check-up hypertransaminasemia (AST 626 U/L; ALT 800 U/L), hyperbilirubinemia (7.4 total; 3.4 conjugated), mild hypoalbuminemia (3.6 g/dl), impaired coagulation (PT 31%; INR 2.6; aPTT 51.5 sec; fibrinogen 151; ATIII 157) were detected. At that time a Clinitest in urines was positive, suggesting the diagnosis of galactosemia. The diagnosis of galactosemia was confirmed by the assay of galactose-1-phosphate uridyltransferase in erythrocytes, showing a highly reduced activity (1.0 U/g Hb; normal values ≥ 2.3). The levels of galactose-1-phosphate in erythrocytes were 9.40 mg/dl (vn: 0.00 – 0.24 mg/dl).

A lactose-free formula was started and continued for the following months, with rapid and complete normalization of the biochemical markers (AST 114 U/L; ALT 316 U/L; total bilirubin 1.9 mg/dl, 0.82 conjugated; PT 93%; INR 1.06; aPTT 34.3 sec; fibrinogen 227; ATIII 127).

## Conclusions

This case emphasizes the importance of thinking to rare metabolic disorders in the differential diagnosis of patients presenting with relatively non-specific symptoms. Particular attention should be paid to the clinical course and to its correlations with dietary changes. In our patient the diagnosis of galactosemia was obscured by the concomitant presence of manifestations suggesting a cow’s milk intolerance.

Galactosemia is an inherited metabolic disorder, due to the deficiency of the enzyme galactose-1-phosphate uridyltransferase and with an estimated prevalence that ranges between 1 in 30,000 and 1 in 60,000 births [1]. The clinical presentations is typically characterized by severe liver involvement, that may progress to fatal liver failure, but manifestations related to the involvement of the gastro-intestinal tract, such as those seen in our patient, are common. The occurrence of sepsis in untreated neonates, mainly due to E.coli, is also frequently reported. Cataract is also a relatively frequent (14%) finding in galactosemia [2]. The diagnosis is based on the identification of clinical and biochemical manifestations of liver disease, on screening tests (clinitest in urines, galactose-1-phosphate in erythrocytes), on the biochemical assay that demonstrates the specific enzymatic defect in blood erythrocytes, and on the molecular analysis of the *GALT* gene.

The treatment of galactosemia is based on the elimination of galactose and galactose-containing foods, that results in complete recovery.

In our patient the presenting symptoms, particularly gastro-intestinal signs such as vomiting on assumption of standard formulas, and the clinical response to repeated dietary changes initially supported the diagnosis of cow’s milk intolerance, a very common disorder in infancy. On the other hand the use of commercial protein hydrolysate formulas, which are generally free from lactose and galactose, prevented the occurrence of clinically overt manifestations of the underlying metabolic disease, until a standard formula was re-introduced at the age of 8 months.

Other factors also contributed to mask the manifestations of galactosemia. In the first few days the presence of biochemical signs of liver disease were erroneously attributed to the maternal use of antiepileptic drugs during pregnancy. It is known that some antiepileptic drugs, such as phenobarbital, fenitoin, carbamazepine, may cross the placental barrier and induce microsomal enzymes in fetal liver. An unexplained liver disease may also be the sign of a broad range of distinct syndromes, including familial hemophagocytosis [3] and complex autoimmune disorders [4,5].

In addition, the absence in the first weeks of life of lens opacities, a typical and peculiar finding in galactosemia, that may raise the suspect of galactosemia, contributed to the delay in achieving the correct diagnosis.

The clinical presentation of galactosemia is characterized by relatively non-specific signs that may be attributed to other disorders. This is a common finding in many metabolic diseases that are characterized in general by complex phenotypes, involving several organs and systems, such as the central nervous system [6,7], the respiratory tract [8-11], the gastro-intestinal tract and liver [12-14], the muscolo-skeletal system [15-18], and the immune system. Such a wide variability and involvement of different systems may be confusing in the diagnostic approach to patients with metabolic disorders.

Simple and widely available screening tests, such as a clinitest inurines, may be useful in patients that assume galactose. The galactose-1-phosphate uridyltransferase assay for newborn screening [19] would prevent misdiagnoses in patients affected by galactosemia. Unfortunately a screening program for this disorder is not available in all countries.

## Competing interests

The authors declare no competing interest.

## Authors’ contribution

RDC, CU, FS, EA and CP followed the patient and made the diagnosis, PV was the consultant hepatologist, GP was the consultant metabolic physician, RDC and GP wrote the paper. All authors read and approved the final manuscript.
